# Overexpression of SMYD3 in Ovarian Cancer is Associated with Ovarian Cancer Proliferation and Apoptosis via Methylating H3K4 and H4K20

**DOI:** 10.7150/jca.29861

**Published:** 2019-07-08

**Authors:** Yahui Jiang, Tianjiao Lyu, Xiaoxia Che, Nan Jia, Qin Li, Weiwei Feng

**Affiliations:** 1 Department of Gynecology, Obstetrics and Gynecology Hospital, Fudan University, 419 Fang Xie Road, Shanghai, 200011. China; 2 Shanghai Key Laboratory of Female Reproductive Endocrine - Related Diseases, Obstetrics and Gynecology Hospital, Fudan University, 419 Fang Xie Road, Shanghai, 200011. China; 3 Department of Gynecology and Obstetrics, Ruijin Hospital,Shanghai Jiaotong University , School of Medicine, 197 Ruijin Road, Shanghai, 200025, China.

**Keywords:** Ovarian cancer, Histone methyltransferase, SMYD3, proliferation

## Abstract

**Background**: Epigenetic regulation has been verified as a key mechanism in tumorigenesis. SET and MYND domain-containing protein 3 (SMYD3), a histone methyltransferase, is a promising epigenetic therapeutic target and is overexpressed in numerous human tumors. SMYD3 can promote oncogenic progression by methylating lysines to integrate cytoplasmic kinase signaling cascades or by methylating histone lysines to regulate specific gene transcription. However, the exact role of SMYD3 in the progression of ovarian cancer is still unknown.

**Methods**: Immunohistochemistry was employed to test SMYD3 expression in ovarian cancer tissues from clinical patients. CCK-8 assay, Real-time cell analysis (RTCA), colony formation assay, cell cycle and apoptosis tested by Flow cytometer were employed to test the effects of SMYD3 on cell proliferation and apoptosis in ovarian cancer cell lines. A PCR array was used to identify the downstream targets of SMYD3. And, PCR and Western blot were used to verify their expression. The binding of SMYD3 on the promoter of target genes were tested by ChIP assays. We also use nude mice subcutaneous tumor model and patient-derived xenograft (PDX) model to investigate the tumor promotive function of SMYD3 *in vivo*.

**Results**: SMYD3 expression was higher in ovarian cancer tissues and cell lines than in normal ovarian epithelial tissue and human ovarian surface epithelial cells (HOSEpiC). After silencing SMYD3, the proliferation of ovarian cancer cells was significantly inhibited *in vitro*. In addition, the SMYD3-specific small-molecule inhibitor BCI-121 suppressed ovarian cancer cell proliferation. Downregulation of SMYD3 led to S phase arrest and increased the cell apoptosis rate. Furthermore, a PCR array revealed that SMYD3 knockdown caused the upregulation of the cyclin-dependent kinase (CDK) inhibitors CDKN2A (p16^INK4^), CDKN2B (p15^INK4B^), CDKN3 and CDC25A, which may be responsible for the S phase arrest. In addition, the upregulation of CD40LG and downregulation of BIRC3 may explain the increased cell apoptosis rate after silencing SMYD3. We also discovered that SMYD3 bound on the promoter of CDKN2A and down-regulated its expression by triple-methylating H4K20. In addition, SMYD3 bound on the promoter of BIRC3 and up-regulated its expression by triple-methylating H3K4. Finally, knocking down SMYD3 could inhibit ovarian cancer growth in nude mice subcutaneous tumor model and PDX model.

**Conclusion**: Our results demonstrated that SMYD3 was overexpressed in ovarian cancer and contributes to the regulation of tumor proliferation and apoptosis via SMYD3-H4K20me3-CDKN2A pathway and SMYD3-H3K4me3-BIRC3 pathway. Thus, SMYD3 is a promising epigenetic therapeutic target for ovarian cancer.

## Introduction

Due to the lack of obvious early symptoms and of an effective diagnosis method, ovarian cancer has become the most fatal gynecological cancer worldwide. Although treatments such as surgery and chemotherapy are utilized, the 5-year survival rate of advanced ovarian cancer remains at approximately 30%-40%.^1^ Therefore, more specific ovarian cancer markers should be discovered and used for early diagnosis or targeted therapy.

Epigenetics refers to the non-Mendelian inheritance of DNA modifications that can influence gene expression. Unlike the genome, the epigenome can be modified by the external environment and can be reversed.^2^ Cancer occurrence and progression rely on both the genome and epigenome, prompting us to pay close attention to the rich epigenetic alterations in cancer.

The underlying epigenetic modifications that can regulate the genes involved in all the hallmarks of cancer include DNA methylation, posttranslational histone modification and small RNA-mediated silencing. However, current epigenetic therapies mainly focus on DNA methylation and histone modification.^3^ Thus, we engaged in the study of histone modifications in cancer.

SET and MYND domain-containing protein 3 (SMYD3) is a histone methyltransferase that has been implicated in cancer pathogenesis and acts as a gene transcriptional regulator. SMYD3 is overexpressed in multiple cancer types, suggesting its essential role in tumor initiation and progression. It can occupy binding motifs on target gene promoters and regulate target gene expression by methylating histones such as H3K4 and H4K5 in the nucleus.^4^, ^5^ In addition, SMYD3 can methylate vascular endothelial growth factor receptor 1 (VEGFR1) and MAP3 kinase 2 (MAP3K2) in the cytoplasm to stimulate downstream signaling.^6^, ^7^ Hence, SMYD3 plays a crucial role in the transcriptional and functional regulation of human carcinogenesis. SMYD3 specifically activates the transcription of a set of downstream genes, including several oncogenes (e.g., N-Myc, Wnt10b, and hTERT), cell cycle genes (e.g., cyclin G1 and CDK2), signal transduction genes (e.g., STAT1 and MAP3K11), thus implicating SMYD3 in cell proliferation, apoptosis, adhesion and migration. SMYD3 has been demonstrated to play an important role in esophageal squamous cell carcinoma, gastric carcinoma, hepatocellular carcinoma, breast carcinoma, etc.^8^ However, whether SMYD3 expression contributes to ovarian cancer development and progression is still unknown.

In this study, we aimed to investigate SMYD3 expression in ovarian cancer and its potential role and regulatory mechanism in cell proliferation and apoptosis. Our study is the first report that SMYD3 is essential for ovarian cancer proliferation and tumorigenesis, indicating the potential of SMYD3 as a new epigenetic therapeutic target for ovarian cancer.

## Materials and Methods

**Human samples:** A tissue array harboring fifty-five ovarian cancer tissues and ten normal ovarian tissues harvested from 2015 to 2017 was obtained from the tissue bank of the Obstetrics and Gynecology Hospital of Fudan University. Ten normal ovarian tissues were obtained from patients who received oophorectomy for non-tumor reasons. Fifty-five epithelial ovarian cancer tissues were collected according to following inclusion criteria: Female, histologically diagnosed to be stage Ⅰ-Ⅳ epithelial ovary primary cancer, no combination of other primary tumors and severe system diseases. Tissues from patients who received new adjuvant chemotherapy or tissues without enough size for making tissue array were excluded. All experiments were approved by the Ethics Committee of the Obstetrics and Gynecology Hospital of Fudan University, and informed consent was obtained from all patients.

**Immunohistochemistry:** Paraffin-embedded sections were deparaffinized, blocked and incubated with SMYD3 antibody (ab187149, 1:100) at 4°C overnight. Horseradish peroxidase-conjugated secondary antibody was added, and the sections were further incubated for 1 h at room temperature, developed using a 3,3'-diaminobenzidine tetrahydrochloride (DAB) substrate kit at room temperature for 1-5 min, and then counterstained with hematoxylin. The extent of immunostaining was quantified using Image-Pro Plus software.

**Cell lines and culture conditions:** The human ovarian epithelial cancer cell line HEY was obtained from Dr. Robert Bast's laboratory at the University of Texas Anderson Cancer Center, Houston, TX. Human ovarian surface epithelial cells (HOSEpiC) and A2780, SKOV3, SKOV3 ip, and OVCAR3 cells were obtained from the Shanghai Key Laboratory of Female Reproduction Endocrine Related Diseases, Obstetrics and Gynecology Hospital, Fudan University. HOSEpiC and all the ovarian epithelial cancer cell lines were routinely maintained in RPMI 1640 medium containing 10% FBS, 100 U/ml penicillin and 100 μg/ml streptomycin and were incubated at 37°C in 5% CO_2_.

**siRNA-mediated gene silencing:** The SMYD3 siRNA duplexes (siRNA-SMYD3: 5'-CCACAAGCGGGAAUGCAAA-3') were synthesized by GenePharma (Shanghai, China). A nontargeting Si-NC (5'-UUCUCCGAACGUGUCACGUdTdT-3') was served as a control. For siRNA transfection, 1×10^5^ cells/well were plated in a 6-well plate. The following day, 5 μl of Lipofectamine 2000 reagent (Invitrogen) was added to 125 μl of Opti-MEM reduced serum medium (Invitrogen) without antibiotics or serum, and the mixture was incubated at room temperature for 5 min (solution A). Then, 50 nM siRNA was added to 125 μl of Opti-MEM without antibiotics or serum (solution B). Solutions A and B were then mixed and incubated at room temperature for 15 min. The cell culture medium was removed, and 250 μl of the Lipofectamine 2000-siRNA mixture and 2 ml of fresh RPMI 1640 medium were added to each well and gently mixed. Forty-eight hours later, the cells were trypsinized for cell counting kit (CCK)-8, cell cycle and apoptosis assays.

**Establishment of a stable SMYD3-knockdown cell line:** The shRNA targeting SMYD3 (sh-SMYD3: GGATGGAGCACCTTCAGAATC) and a negative control with a scrambled sequence (sh-NC: TTCTCCGAACGTGTCACGT) were designed and constructed by GENECHEM (Shanghai, China) using a hU6-MCS-Ubiquitin-EGFP-IRES-puromycin lentivirus vector. Lentiviruses were produced by the transfection of 293T cells with plasmids using packaging mix (GENECHEM). HEY and A2780 cells were transfected and selected in puromycin according to the manufacturer`s instructions.

**cDNA synthesis and SYBR green real-time PCR:** Total RNA was extracted from treated and control cells using TRIzol reagent (Invitrogen) according to the manufacturer's instructions. Reverse transcription was performed using a TaKaRa reverse transcription kit (RR036A). Semiquantitative RT-PCR was performed by using SYBR Green Master Mix (Takara, DRR820A) and a Real-time PCR System. The relative fold changes were calculated using the ΔΔCt method. The sequences of the specific primers are listed in Supplemental Table 1(Table S1).

**Western blotting**: Antibodies against SMYD3 (ab187149), CDKN2A (ab108349), CDKN2B (affbiotech, AF0230), CDKN3 (affbiotech, DF8911), CDC25A (affbiotech, AF6252), BIRC3 (ab32059), CD40LG (ab52750) and GAPDH (ARG65680) were used for western blotting. Cells and tissues were lysed in RIPA buffer containing 1:100 PMSF. Protein concentrations were measured by the BCA Protein Assay. Equal amounts of protein were resolved by SDS-PAGE and transferred to PVDF membranes, which were incubated with appropriate primary antibodies at the indicated concentrations. Immune complexes were detected with HRP-conjugated secondary antibodies (ARG65351) and ECL reagent (Millipore, WBKLS0500).

**CCK-8 assays:** Cell viability was evaluated by CCK-8 (Dojindo, JE603). Cells were transfected with siRNA-SMYD3 or nontargeting siRNA using Lipofectamine 2000 reagent. After a 24h incubation, cells were plated on 96-well plates at 5×10^3^ cells/well and incubated for 72 h at 37°C. CCK-8 was added at 24h, 48h and 72h, and the plates were incubated for 1 h at 37°C. The absorbance was measured at 450 nm using a BioTeK Reader.

**Real-time analysis of cell proliferation:** Real-time cell analysis (RTCA) experiments were carried out using the xCELLigence RTCA DP instrument. Briefly, 5×10^3^ cells were inoculated in 96-well microtiter E-plates for the cell proliferation assay. The slope indicates the increase in cell impedance per unit time (slope=impedance/time). The median and SD were calculated from 3 individual replicate wells.

**Cell cycle distribution:** Cells were fixed with 500 μl of ice-cold 70% ethanol and treated with 1 mg/mL RNAse for 30 min. Intracellular DNA was labeled by incubation with propidium iodide (50 μg/mL) at 4°C in the dark. The samples were then analyzed using a flow cytometer (FACSVantage SE; BD Biosciences) to detect differences in cell cycle distribution after SMYD3 silencing.

**Apoptosis assays:** Cells were stained with 200 μl of Annexin V-fluorescein iso-thiocyanate (BD 556547) at 4°C for 15 min and then with 1 mL of propidium iodide for 5 min. The proportion of apoptotic cells was determined by flow cytometry.

**Colony formation assays:** The cells were seeded in 6-well plates at a density of 800 cells/well. After incubation for 12 days, the colonies were fixed with 4% formaldehyde for 15 min and stained with 2% crystal violet for 20 min. The number of colonies containing >50 cells was calculated.

**PCR array:** An RT2 Profiler Custom PCR array was used to simultaneously examine the mRNA levels of 114 genes, including five housekeeping genes of HEY-NC and HEY-shSMDY3 in 384 well plates according to the protocol of the manufacturer (BioTNT,Shanghai,China). Real-time PCR was performed using the RT2 SYBR green qPCR Master Mix (SABiosciences) on an ABI VIIA 7 Fast 384-well realtime PCR machine (Applied Biosystems, Foster City, CA, USA). Results were analyzed using the PCR Array Data Analysis Web Portal (SABiosciences). Glyceraldehyde-3-phosphate dehydrogenase (GAPDH) and β-actin were chosen as internal loading controls for standardization between samples and relative mRNA levels of target genes were calculated by the 2-ΔΔ Ct method.

**Immunofluorescence:** The cells were grown on sterile coverslips, fixed with 4% paraformaldehyde and permeabilized using 0.1% Triton X-100. Cells were incubated with the SMYD3 antibody and then the rhodamine-conjugated anti-rabbit secondary antibody. Finally, the cells were stained with 4',6-diamidino-2-phenylindole (DAPI) and observed with confocal microscopy.

**Chromatin immunoprecipitation assays:** Cells were subjected to chromatin immunoprecipitation based on an EZ-ChIP protocol (Millipore, 17-371). Briefly, chromatin and DNA were cross-linked by treatment with 37% formaldehyde. Cell lysates were collected and sonicated to shear DNA. Soluble chromatin was incubated for 2 hrs at 4°C with 60 μl of protein A-agarose-salmon sperm DNA. Pre-cleared lysate was incubated overnight at 4°C with 1 μg of normal mouse IgG (as negative control), 5 μg of anti-SMYD3 (ab85277), 5 μg of anti-H4K20me3 (ab177190) or 5 μg of anti-H3K4me3 (ab8580). The antibody-protein-DNA complexes were precipitated with 60 μl of protein A-agarose beads at 4°C for 2 hrs. Complexes were eluted in elution buffer (0.1 mM NaHCO3 and 1% SDS) before reversal of cross-links overnight at 65°C under high salt conditions (0.5 M NaCl). After proteinase K digestion, DNA was extracted in 25:24:1 phenol/chloroform/isoamyl alcohol and precipitated overnight in ethanol at -20°C, and DNA was then eluted in Tris/EDTA buffer. We calculated △ct (△ct=ct(target gene)-ct(negative control)-ct(input)), and did statistical analysis by 2^-△△ct^ .The presence of CDKN2A and BIRC3 gene promoter sequences in immunoprecipitated DNA was identified by realtime-PCR using the primer sequences listed in Supplemental Table 2 (Table S2). All experiments were repeated at least three times.

**Animal xenografts:** Ten specific pathogen-free female BALB/c nu/nu mice (4 weeks old) were randomly divided into the experimental group (HEY-shSMYD3) and the control group (HEY-NC). HEY-NC and HEY-shSMYD3 cells (1.5×10^6^) were harvested by centrifugation, and the cell suspensions (200 μl) were subcutaneously injected into the right flank of mice in the experimental and control groups. The maximum diameter (a) and minimum diameter (b) were measured using a Vernier caliper every 3 days beginning on the 12th day, and the tumor volume (V) was calculated according to the following formula: V=0.5×a×b^2^. After 3 weeks of *in vivo* tumor growth, the mice were sacrificed, and the tumors were removed and weighed using an electronic balance. All experimental procedures were approved by the Ethics Committee of the Obstetrics and Gynecology Hospital of Fudan University.

### Patient-Derived Xenograft (PDX) model establishment and SMYD3 inhibition

We collected the primary tumor tissue of a stage Ⅲ ovarian high-grade seromucinous carcinoma patient and implanted it into the omentum majus of NOD/SCID mice to build the first generation of PDX model. After subculture, we chose tumor tissues from the F3 generation which had higher inheritable stability and equally implanted into the omentum majus of twelve 4-week NOD/SCID mice. Then, we injected lentivirus encoding an siRNA directed against SMYD3 (GGATGGAGCACCTTCAGAATC) or scrambled sequence RNAs (TTCTCCGAACGTGTCACGT) intraperitoneally at the dose of 2×10^6^ TU on days 1, 3, 7, 10, 14, 21, 28, 35, 42 and 49 into NOD/SCID mice. All mice were euthanized after 54 days, and then the tumors were collected for subsequent protein expression analysis.

**Statistical analysis:** Statistical analysis was conducted using SPSS version 15.0 (SPSS, Chicago, IL, USA). Student's t tests were used for the analysis of categorical data, and all statistical tests were 2-sided. A P value less than 0.05 indicated statistical significance.

## Results

### SMYD3 was overexpressed in ovarian cancer compared with human normal ovarian surface epithelium

**Figure 1:**
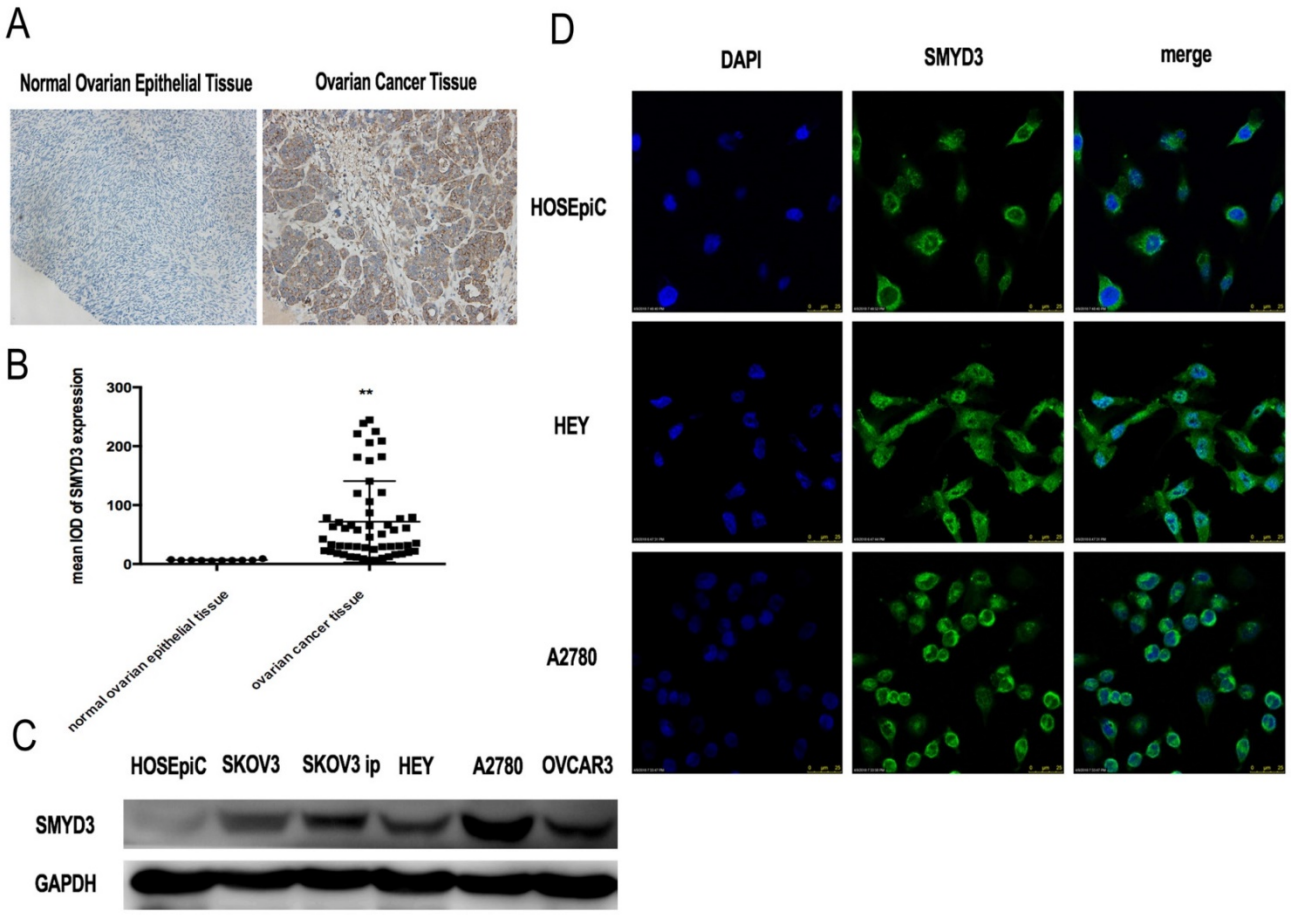
** SMYD3 is overexpressed in human ovarian cancer.** (A) Immunohistochemical staining of SMYD3 expression in human normal ovarian epithelial tissue and ovarian cancer tissue. (B) Mean IOD (integrated optical density) of SMYD3 after immunohistochemical staining of human normal ovarian epithelial tissue and ovarian cancer tissue analyzed by Image-Pro Plus. (C) SMYD3 protein expression level in human ovarian surface epithelial cells (HOSEpiC) and human ovarian cancer cell lines. (D) Immunofluorescence staining of SMYD3 in HEY and A2780 cells.

We analyzed SMYD3 expression in normal ovarian surface epithelium from 10 women and ovarian cancer tissues from 55 women by immunohistochemistry. The pathological diagnoses of these 55 ovarian cancer patients were all high-grade ovarian serous adenocarcinoma. The average age of the patients was 55.40±10.62 years (range 34~75 years). Five cases were in stageⅠ, 8 cases in stageⅡ,36 cases in stage Ⅲ,6 cases in stage Ⅳ. The immunohistochemistry results showed that SMYD3 expression was significantly higher in ovarian cancer tissues than in normal ovarian surface epithelium (Figure 1A, 1B). Then, we investigated SMYD3 expression in ovarian cancer cell lines and HOSEpiC. As shown in Figure 1C, SMYD3 expression was lowest in HOSEpiC and highest in A2780 cells. SMYD3 was also highly expressed in the other ovarian carcinoma cell lines. Therefore, increased SMYD3 expression is correlated with ovarian carcinogenesis.

### SMYD3 was primarily expressed in the cytoplasm of normal ovarian surface epithelial cells and was differentially expressed in various ovarian cancer cell lines

We chose the ovarian cancer cell lines HEY and A2780 to further investigate the localization of SMYD3 compared with those in HOSEpiC via a cell immunofluorescence assay, which showed that SMYD3 was predominantly localized in the cytoplasm in A2780 cells and HOSEpiC. In HEY cells, SMYD3 was localized in both the cytoplasm and nucleus, with greater expression in the cytoplasm (Figure 1D).

### The proliferation of ovarian cancer cell lines was inhibited and cell cycle was arrested at S phase after SMYD3 knockdown

**Figure 2:**
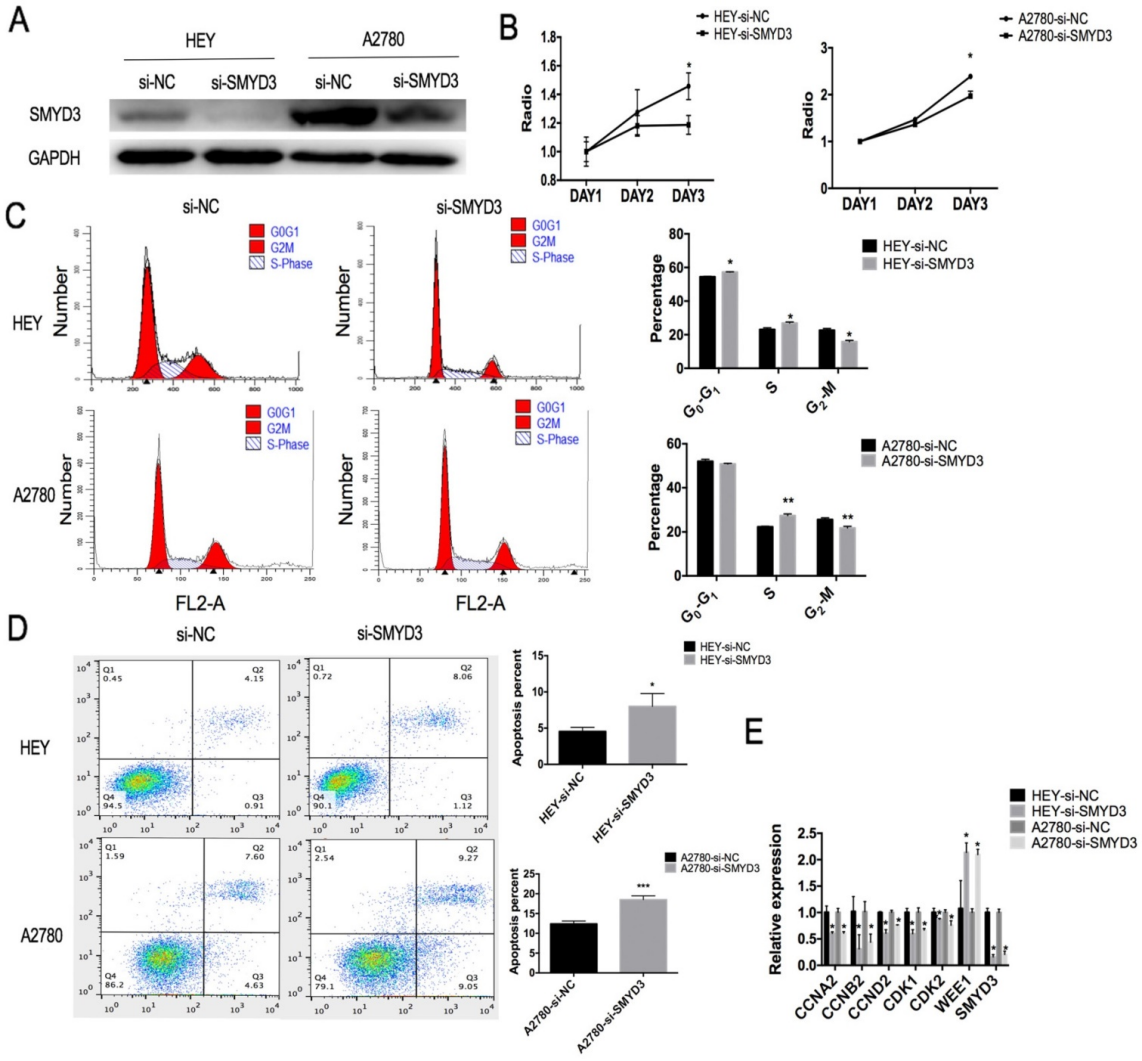
** The proliferation and apoptosis of ovarian cancer cell lines are affected by siRNA-mediated downregulation of SMYD3.** (A) The transfection efficiency of siRNA targeting SMYD3 was analyzed by western blot. (B) Cell proliferation after SMYD3 silencing in the HEY and A2780 cell lines was determined using a CCK-8 assay. Cell cycle distribution (C) and cell apoptosis (D) in the si-SMYD3 and si-NC groups were determined by flow cytometry. (E) Changes in the mRNA expression of cell cycle-related genes in HEY and A2780 cells after silencing SMYD3.

In order to study the effect of SMYD3 on ovarian cancer cell line proliferation, siRNAs were constructed to knockdown SMYD3. We detected the interference efficiency of this siRNA by real-time PCR and selected the most efficient siRNA to knockdown SMYD3 expression in HEY and A2780 cells (Figure 2A). The CCK-8 assay showed that the negative control (si-NC) cells grew more rapidly than the si-SMYD3 cells (Figure 2B). Then, we used flow cytometry to investigate alterations in the cell cycle after SMYD3 knockdown. We found an increased percentage of cells in S phase and a decreased percentage of cells in G_2_/M phase after SMYD3 knockdown in both the HEY and A2780 cell lines, while more cells were in G_0_/G_1_ phase in HEY cell line only after SMYD3 knockdown (Figure 2C). Therefore, silencing SMYD3 may cause S phase arrest and lead to decreased proliferation.

Then, we examined potential differences in cell cycle-related gene expression between the si-NC and si-SMYD3 groups in the HEY and A2780 cell lines. Using real-time PCR, we found that silencing SMYD3 significantly decreased the mRNA levels of CCNA2, CCNB2, CCND2, CDK1 and CDK2 (p<0.05) but increased the mRNA levels of WEE1 (p<0.05), suggesting an essential role for SMYD3 in ovarian carcinoma proliferation (Figure 2E).

### Silencing SMYD3 induced ovarian cancer cell apoptosis

By flow cytometry, we found that silencing SMYD3 increased the percentage of late apoptotic cells in the HEY cell line (p<0.05) and the percentages of early and late apoptotic cells in the A2780 cell line (p<0.05) (Figure 2D).

### Establishment of a stable SMYD3-knockdown cell lines

**Figure 3:**
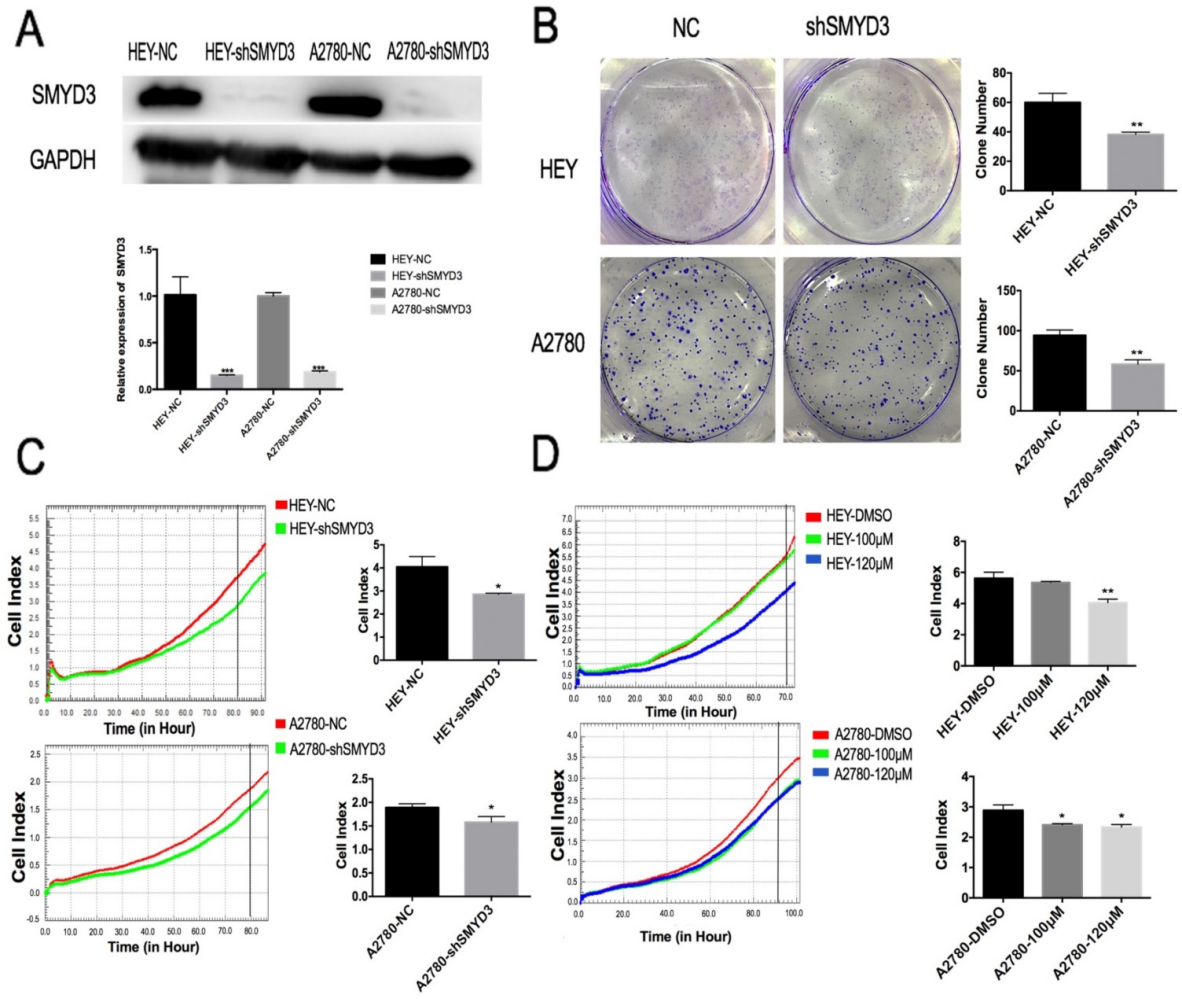
** Establishment of stable SMYD3-knockdown cell lines and validation of functional changes in proliferation and apoptosis.** (A) The infection efficiency of lentivirus carrying shRNA targeting SMYD3 was examined by western blotting and real-time PCR. (B-C) Cell proliferation following SMYD3 knockdown in the HEY and A2780 cell lines was determined using colony formation assays (B) and RTCA (C). (D) Changes in HEY and A2780 cell proliferation following treatment with the SMYD3 inhibitor BCI-121.

To further explore the function and downstream molecules of SMYD3, we established stable HEY and A2780 cell lines that constitutively expressed shRNA targeting SMYD3 (HEY-shSMYD3 and A2780-shSMYD3 cells). The infection efficiency was examined by real-time PCR and western blot (Figure 3A). The relative SMYD3 mRNA expression level was knocked down to 0.13±0.032 and 0.17±0.01 in HEY-shSMYD3 and A2780-shSMYD3 cells compared with the negative control cells (HEY-NC and A2780-NC), respectively. In addition, at the protein level, SMYD3 was knocked down by 95% and 88% in HEY-shSMYD3 and A2780-shSMYD3 cells, respectively. Therefore, we successfully established two stable SMYD3-knockdown cell lines, HEY-shSMYD3 and A2780-shSMYD3.

We then performed RTCA and plate colony formation assays to validate the effect of SMYD3 on ovarian cancer cell proliferation using stable SMYD3-knockdown cell lines. In the HEY cell line, the proliferation of HEY-shSMYD3 cells was obviously inhibited compared with that of HEY-NC cells (p<0.05) as examined by RTCA and plate colony formation assay (p<0.01). The A2780 cell line showed the same phenomenon after SMYD3 knockdown (Figure 3B and 3C).

### The specific small-molecule inhibitor of SMYD3 suppressed ovarian cancer proliferation

BCI-121 is a new SMYD3 small-molecule inhibitor that can significantly reduce SMYD3 activity. It can compete with histones for binding to SMYD3, so the functional readout of its activity is a reduction in target gene mRNA expression. BCI-121 can also inhibit SMYD3 function in the cytoplasm by downregulating p-ERK levels.^9^ We pretreated HEY and A2780 cells in complete culture medium containing 100 μM or 120 μM BCI-121 for 48 h. Then, the cells were plated in an RTCA plate to monitor proliferation in real time. After more than 70 h of observation, BCI-121 was shown to successfully inhibit HEY and A2780 cell proliferation, but the dose-dependent effect was not obvious.

### Effects of SMYD3 on cell cycle checkpoint genes and apoptosis genes correlated with ovarian carcinoma progression

**Figure 4:**
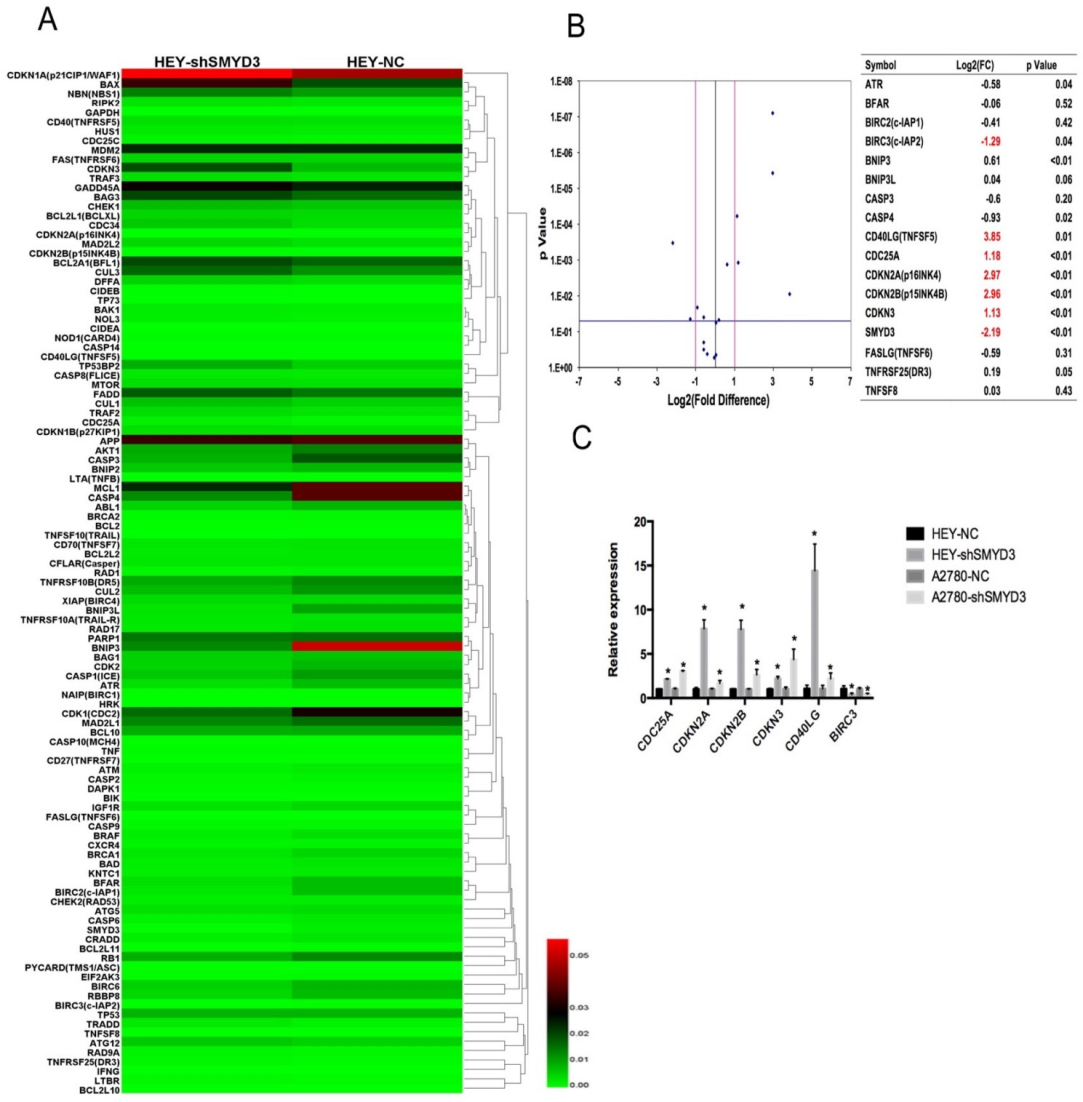
** Tumor cell cycle checkpoint and apoptosis gene expression levels in HEY cells screened by PCR array.** (A) Heat map results showing the mRNA expression of all 109 genes involved in the cell cycle checkpoint and apoptosis pathway after SMYD3 knockdown. (B) The volcano plot shows the validation results of 16 genes among all 109 genes whose expression changed by more than 2-fold by real-time PCR in HEY-NC and HEY-shSMYD3 cells. The table and plot show that only 6 genes (CDKN2A, CDKN2B, CDKN3, CDC25A, CD40LG, and BIRC3) had expression changes of greater than 2-fold after SMYD3 was knocked down. (C) The mRNA expression changes in the above 6 genes in the HEY and A2780 cell lines after SMYD3 knockdown.

**Figure 5:**
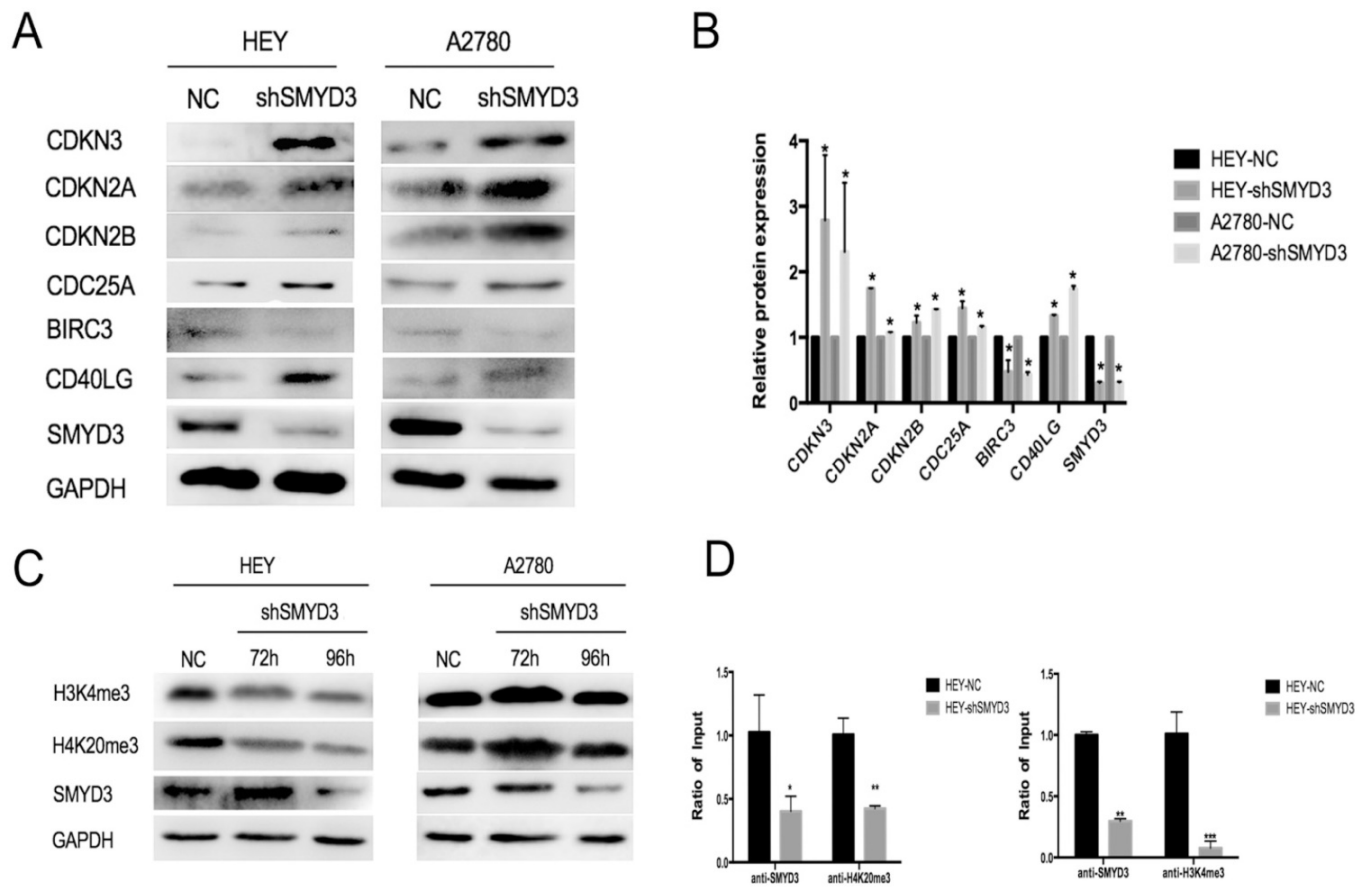
** SMYD3 regulated ovarian cancer proliferation and apoptosis though SMYD3-H4K20me3-CDKN2A and SMYD3-H3K4me3-BIRC3, respectively.** (A-B) The protein expression changes in the above 6 genes in the HEY and A2780 cell lines after SMYD3 knockdown in HEY and A2780 cells. (C) The protein expression changes of H3K4me3 and H4K20me3 after silencing SMYD3 in HEY and A2780 cells. (D) SMYD3, H4K20me3 at the CDKN2A promoter and SMYD3, H3K4me3 at the BIRC3 promoter were analyzed by CHIP assay in HEY-NC and HEY-shSMYD3. Levels were expressed as the ratio of the signal intensity of the immunoprecipitation product to the input (see Methods).

Since we found that knockdown of SMYD3 inhibited cell proliferation, arrested cells in S phase and induced apoptosis, 109 genes involved in cell cycle checkpoints and apoptosis were examined using a focused PCR array approach in HEY-NC and HEY-shSMYD3 cells. Seventeen genes were differentially expressed by at least 2-fold in HEY-shSMYD3 cells compared with HEY-NC cells, among which 12 were downregulated and 8 were upregulated (Figure 4A). Then, we used real-time PCR to confirm the changes in the expression of these 17 genes in HEY-NC and HEY-shSMYD3 cells. The volcano plot (Figure 4B) revealed that only 6 genes (CDKN2A, CDKN2B, CDKN3, CDC25A, CD40LG, and BIRC3) showed expression changes of at least 2-fold after SMYD3 knockdown. Only BIRC3 was significantly downregulated, while CDKN2A, CDKN2B, CDKN3, CDC25A, and CD40LG were notably upregulated. The mRNA expression levels of these 6 genes were validated again in the A2780-NC and A2780-shSMYD3 cell lines, in which they exhibited the same trends as in HEY cells (Figure 4C). Then we examined protein expression alteration after knocking down SMYD3 in HEY and A2780 cell lines via western blot, and the results were consistent with mRNA expression levels as above (Figure 5A,5B).

CDKN2A, CDKN2B, CDKN3 and CDC25A are cell cycle-related genes. Among them, CDKN2A, CDKN2B, and CDKN3 are CDK inhibitors that can cause cell cycle arrest and CDK2 downregulation.

Among apoptosis-related genes, CD40LG was upregulated and BIRC3 was downregulated after SMYD3 abrogation, and these events may be responsible for the increased apoptosis rate.

### SMYD3 regulated target genes expression by triple-methylating H3K4 and H4K20

Since SMYD3 is a histone methyltransferase, and it is well-known that SMYD3 regulate target genes expression by binding to gene`s promoter and catalyze histone lysine methylation, we used CHIP assay to further investigate whether SMYD3 regulate above 6 genes by histone lysine methylation in HEY and A2780 cell line. On the basis of published paper, SMYD3 usually promotes gene expression by triple-methylating histone3 lysine-4(H3K4) and inhibits gene expression by triple-methylating histone4 lysine-20(H4K20). Via CHIP assay, we found a greater SMYD3- and H3K4me3-binding to BIRC3 promoter in HEY-NC cells than in HEY-shSMYD3 cells when the amount of DNA immunoprecipitated was normalized by the amount of the amplified DNA in the corresponding non-immunoprecipitated chromatin solution. We also found a greater SMYD3- and H4K20me3-binding to CDKN2A promoter in HEY-NC cells than in HEY-shSMYD3 cells (Figure 5D). What` s more, the target promoter regions of BIRC3 and CDKN2A including SMYD3 binding site CCCTCC/GGAGGG. However, there was no specific binding between SMYD3 and 6 target genes above found in A2780 cell line. Furthermore, we transfected HEY and A2780 using shRNA targeting SMYD3 and collected the protein after 72 and 96 hours. As Figure 5C shows, the protein expression level of H3K4me3 and H4K20me3 was decreased when SMYD3 was downregulated in HEY cells, but there was no change of protein expression level of H3K4me3 and H4K20me3 in A2780 cells. This phenomenon could be explained by immunofluorescence assay in Figure 1C that SMYD3 only localized in cytoplasm of A2780 cells, but localized both in nucleus and cytoplasm in HEY cells. To sum up, SMYD3 could inhibit CDKN2A expression by triple-methylating H4K20 and promote BIRC3 expression by triple-methylating H3K4.

### Knockdown of SMYD3 inhibited ovarian cancer proliferation *in vivo*

**Figure 6:**
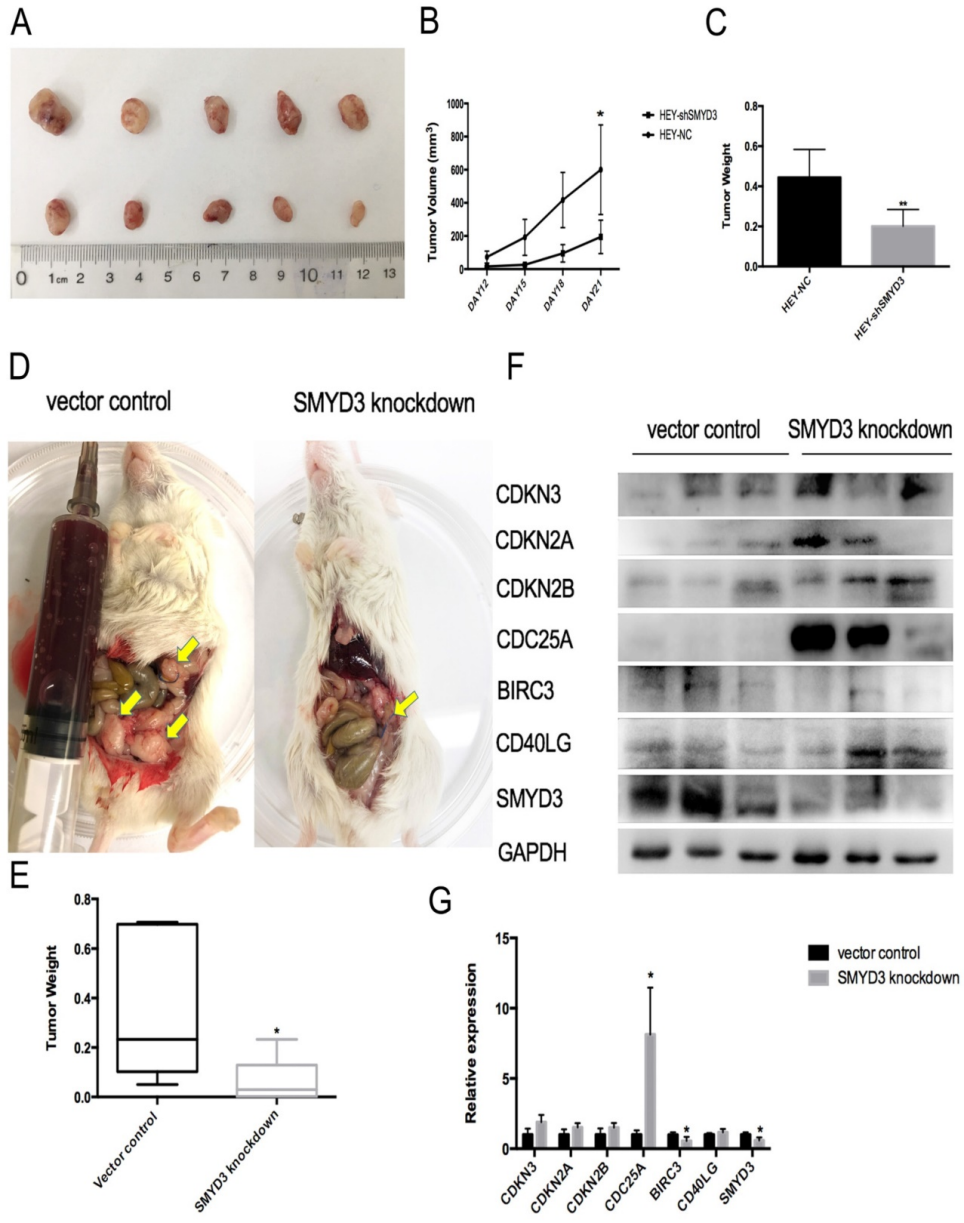
** Knockdown of SMYD3 inhibits ovarian cancer carcinogenesis *in vivo*.** (A) HEY-NC and HEY-shSMYD3 cell xenografted tumors were collected and photographed. The tumor tissues in the first line belonged to HEY-NC group, and the second line belonged to HEY-shSMYD3 group.(B) Tumor growth curves in nude mice in the HEY-NC and HEY-shSMYD3 groups. (C) Tumors from the HEY-NC and HEY-shSMYD3 groups were weighed in the end of the experiment. (D) Dissection of NOD/SCID mice in vector control group and SMYD3 knockdown group in the 54^th^ days after patient ovarian cancer tumors implantation. The arrows pointed to new tumor lesions inside the mice. (E) Tumors from vector control group and SMYD3 knockdown group were collected and weighed. (F-G) The protein expression of SMYD3 and its downstream regulators CDKN3, CDKN2A, CDKN2B, CDC25A, BIRC3, CD40LG in the tumor tissues of vector control group and SMYD3 knockdown group.

HEY-shSMYD3 and HEY-NC cells were injected subcutaneously into the flanks of nude mice. We measured tumor size every 3 days beginning on the twelfth day, and we found that the tumors in the HEY-NC group grew more rapidly than those in the HEY-shSMYD3 group. On the 21st day, we sacrificed all the nude mice and weighed the tumors. As shown in Figure 6, the tumors in the HEY-shSMYD3 group were smaller and lighter than those in the HEY-NC group (p<0.01). These results revealed that SMYD3 promoted ovarian cancer cell proliferation *in vivo*.

In order to investigate the effect of SMYD3 inhibition on human epithelial ovarian cancer repression, we established ovarian cancer PDX model with human ovarian cancer tissue. Twelve F3 generation tumor-bearing mice were equally divided into two groups. We intraperitoneally injected lentivirus encoding an siRNA directed against SMYD3 (SMYD3 knockdown group) or scrambled sequence RNAs (vector control group) into these two groups respectively. 54 days later, we sacrificed the mice and collected tumors from these two group. 50% of mice in SMYD3 knockdown group formed tumor (3/6) and all the mice in vector control group formed tumor (6/6). As Figure 6D and 6E showed that the tumor weight of SMYD3 knockdown group (0.08±0.08g) were smaller than vector control group (0.37±0.27g) (p=0.04). Then we used western blot to compare protein expression of SMYD3 and its downstream regulators in tumor tissues of both groups. SMYD3 was successfully knocked-down in tumor of SMYD3 knockdown group (p=0.04). CDC25A was significantly overexpressed (p=0.02) and BIRC3 was significantly down-regulated (p=0.02) after SMYD3 knockdown in ovarian cancer PDX model. From Figure 6F, we could figure out that 2 out of 3 tumor tissues in SMYD3 knockdown group had higher expression of CDKN2A, CDKN2B, CDKN3 and CD40LG than vector control group. Therefore, SMYD3 can repress ovarian cancer progression both in nude mice model and PDX model.

## Discussions

Ovarian cancer has a high morbidity rate among gynecological cancers, and patients are often diagnosed at an advanced stage.^10^ We found higher SMYD3 expression in ovarian cancer tissues and cell lines than in normal ovarian epithelial tissues and HOSEpiC, respectively. These data indicated that SMYD3 plays a role in ovarian cancer carcinogenesis.

SMYD3 is highly expressed in many cancer cells and can promote cancer development and progression by regulating tumor proliferation, apoptosis, invasion and metastasis.^8^ SMYD3 has different functions in different subcellular locations. In liver cancer and colon cancer, SMYD3 is localized in the cell nucleus and selectively promotes oncogene expression via interplay with histones. However, in lung cancer and pancreatic cancer, SMYD3 is localized in the cytoplasm and activates the ERK pathway by methylating MAP3K2.^7^, ^11^-^13^ Although SMYD3 is tightly connected to carcinogenesis, there are few articles about the role of SMYD3 in ovarian cancer.

In our previous study, we found that the proliferation of the SMYD3-knockdown HEY cell line was obviously inhibited compared with that of the negative control cells. Therefore, we selected another ovarian cell line, A2780, which had the highest SMYD3 expression among all ovarian cell lines tested to further investigate the role of SMYD3 in promoting ovarian cancer proliferation. As shown previously, SMYD3 can promote HEY and A2780 cell proliferation by accelerating S phase. Then, we examined the changes in cyclin and CDKs after silencing SMYD3 to further confirm its role in cell cycle alterations. At the mRNA level, CCNA2, CCNB2, CCND2, CDK1 and CDK2 levels were obviously decreased, and WEE1 levels were increased. Christopher C. Porter et al. reported that the inhibition of WEE1 prevents cytarabine-induced S phase arrest in acute myeloid leukemia.^14^ Moreover, Wei et al. demonstrated that WEE1 degradation contributes to Smurf1-regulated S phase progression.^15^ Therefore, WEE1 upregulation may explain the S phase arrest after SMYD3 inhibition. CCNA2 and CDK2 are key regulatory proteins that promote the transition from S phase to G_2_/M phase.^16^ Downregulation of CCNB2 prevents S phase exit, which may lead to S phase arrest.^17^ Similarly, CDK1 and CCND2 have been reported to play a role in S phase arrest.^18^, ^19^.

To further investigate the mechanism underlying the observed phenomenon, we performed a PCR array and compared the expression of several cell cycle-related genes between HEY-NC and HEY-shSMYD3 cells. The CDK inhibitors CDKN2A (p16^INK4^), CDKN2B (p15^INK4B^), CDKN3 and CDC25A were overexpressed by more than 2-fold when SMYD3 was knocked down. Wang et al. reported that 3-hydroxyterphenyllin causes S phase arrest in the ovarian cancer cell lines A2780/CP70 and OVCAR3 by downregulating CDK2 and CDK4 and upregulating CDC25A and cyclin B1.^20^ CDKN3 prevents the activation of CDK2 by dephosphorylating Thr^160^, thus inhibiting the full activation of cyclin E-CDK2 and cyclin A1-CDK2, which play essential roles in the initiation and progression of S phase.^21^, ^22^ High CDKN2A expression has been reported to downregulate CDK2 activity by triggering the reassortment of cyclin-CDK inhibitor complexes,^23^, ^24^ which can induce S phase arrest in cancer cells. In addition, CDKN2A and CDKN2B are members of the INK4 family that interact specifically with CDK4/6 and inhibit its function. Although CDK4/6 mainly leads to G_1_ phase arrest, Wolter F et al. reported that decreased CDK4 expression can also cause S phase arrest.^25^, ^26^.

In addition, we found that BIRC3 expression was reduced and that CD40LG expression was significantly increased after downregulating SMYD3, which could explain the increased apoptosis rate after silencing SMYD3. High expression of the inhibitor of apoptosis (IAP) family member BIRC3 (cIAP2) is associated with many cancers because it can protect against various endogenous and exogenous apoptotic triggers.^27^, ^28^ Therefore, low BIRC3 expression after silencing SMYD3 can induce cell apoptosis. In addition, CD40LG, which is also known as CD40L and TNFSF5, also contributes to increased cell apoptosis. CD40 is a type I transmembrane protein belonging to the tumor necrosis factor (TNF) receptor family. The binding of CD40L, a type II transmembrane protein expressed mainly but not only in activated T cells, plays an important role in immune regulation and cell death.^29^ High CD40 expression levels can be detected in a wide range of human carcinomas, including ovary, liver, lung, cervix bladder and breast cancer. The stimulation of CD40 on cancer cells by recombinant soluble (rs) CD40L can suppress cell proliferation and induce apoptosis *in vitro* and *in vivo*.^30^, ^31^ Bernd Koppold et al. reported that the transduction of tumor cells with recombinant adeno-associated virus (rAAV) encoding CD40L results in the strong activation of dendritic cells; these data helped the overexpression of CD40L become a promising strategy for tumor immunotherapy.^32^ When we knocked down SMYD3, CD40L expression increased by more than 10-fold in the HEY cell line. This finding implies that SMYD3 may be a promising target for cancer immunotherapy.

We firstly built nude mice subcutaneous tumor model by injecting human ovarian cancer cell line subcutaneously to test the function of SMYD3 *in vivo*. Since patient`s ovarian cancer tissue provided a better simulation environment for the progression of ovarian cancer than cell line, we further established a PDX model. This model also verified the changes of CDKN3, CDKN2A, CDKN2B, CDC25A, CD40LG and BIRC3 expression after SMYD3 knocking down.

As a histone methyltransferase, SMYD3 had been repeatedly reported that promotes target genes expression by binding to its promoter and methylating H3K4 in multiple cancers. For example, Hamamoto R found that SMYD3 upregulated WNT10B by triple-methylating H3K4 in breast cancer.^33^ In addition, SMYD3 could decrease gene expression by methylating H4K20 as well. Vieira FQ announced that SMYD3 could regulate CCND2 expression though H4K20me3 in prostate cancer.^34^ Therefore, we further explored the mechanism how SMYD3 regulated tumor-related gene in ovarian cancer. We discovered that SMYD3 could promote BIRC3 expression though H3K4me3 and inhibit CDKN2A expression though H4K20me3. Thus, SMYD3 contributed to a more aggressive phenotype of ovarian cancer.

SMYD3 can also regulate gene expression in cytoplasm by methylating lysines of MAP3K2, VEGFR1 and AKT^6^, ^7^, ^35^. In our study, we found SMYD3 expressed in cytoplasm and nucleus in HEY cells, but expressed only in cytoplasm in A2780 cells. After silencing SMYD3, there was no alteration in protein expression of H3K4me3 and H4K20me3 in A2780 cells. We also could not found SMYD3, H3K4me3 or H4K20me3-enrichment on the promoter of CDKN2A and BIRC3 by CHIP assay in A2780 cells. Therefore, SMYD3 putatively regulated A2780 cells tumor- related gene expression by lysine methylation of certain important signal molecule in cytoplasm.

In conclusion, we demonstrated that SMYD3 is overexpressed in ovarian cancer compared with normal ovarian epithelial tissue. SMYD3 promotes ovarian cancer proliferation and interferes with apoptosis *in vitro* and *in vivo* by regulating cell cycle checkpoints and apoptosis-related proteins though SMYD3-H4K20me3-CDKN2A pathway and SMYD3-H3K4me3-BIRC3 pathway. Furthermore, the specific SMYD3 small-molecule inhibitor BCI-121 can effectively inhibit the proliferation of ovarian cancer cells. These data indicated that SMYD3 is a promising epigenetic therapeutic target for ovarian cancer.

## Supplementary Material

Supplementary tables.

## Abbreviations

SMYD3: SET and MYND domain-containing protein 3
PDX: patient-derived xenograft
CCK-8: cell counting kit-8
RTCA: real-time cell analysis
PCR: polymerase chain reaction
ChIP: Chromatin immunoprecipitation
HOSEpiC: human ovarian surface epithelial cell
CDK: cyclin-dependent kinase
H3K4: histone3 lysine-4
H4K20: histone4 lysine-20
VEGFR: vascular endothelial growth factor receptor
IOD: integrated optical density
CDKN2A: cyclin dependent kinase inhibitor 2A
CDKN2B: cyclin dependent kinase inhibitor 2B
CDKN3: cyclin dependent kinase inhibitor 3
CDC25A: cell division cycle 25A
BIRC3: baculoviral IAP repeat containing 3
CD40LG: CD40 ligand
CCNA2: cyclin A2
CCNB2: cyclin B2
CCND2: cyclin D2
CDK1: cyclin dependent kinase 1
CDK2: cyclin dependent kinase 2
