# Supplementary material for: Overexpression of SMYD3 in Ovarian Cancer is Associated with Ovarian Cancer Proliferation and Apoptosis via Methylating H3K4 and H4K20
Source: J Cancer. 2019 Jul 8;10(17):4072–84. doi: 10.7150/jca.29861 (PMC6692630; doi:10.7150/jca.29861)
Supplement: Supplementary file 1 — Supplementary tables. [file jcav10p4072s1.pdf]

Supplemental Table 1: Primers for RT-PCR

Gene	Primer sequences (forward)	Primer sequences (reverse)
CCNA2	CTGGTGGTCTGTGTTCTGTGA	TCTTGGATGCCAGTCTTACTCA
CCNB2	CGACGGTGTCCAGTGATT	AGCCAAGAGCAGAGCAGT
CCND2	ATCACCAACACAGACGTGGA	TGCAGGCTATTGAGGAGCA
CDK1	TAGCGCGGATCTACCATACC	CATGGCTACCACTTGACCTGT
CDK2	TCCAGGATGTGACCAAGCC	CTGAGTCCAAATAGCCCAAGG
WEE1	GGCTCTGTTGATGAGCAGAACGCTT	CTCAAGCCTCGGCGGCCAACTTGC
CDKN2A	GCTGCCCAACGCACCGAATA	ACCACCAGCGTGTCCAGGAA
CDKN2B	AGTCAACCGTTTCGGGAGGCG	ACCACCAGCGTGTCCAGGAAG
CDKN3	ATCACCCATCATCATCCAATCG	TCTCCCAAGTCCTCCATAGCAG
CDC25A	AGAACCCTATTGTGCCTACTG	TACTCATTGCCGAGCCTATC
CD40LG	CTCTTCCCTCCCCCAGTCTC	AAGAAGAGAACTGACTAGCAACG
GAPDH	GGGAAGGTGAAGGTCGGAGT	GGGGTCATTGATGGCAACA
BIRC3	CTTTGCCTGTGGTGGAAAAT	ACTTGCAAGCTGCTCAGGAT
SMYD3	GGGCCCACCTCTTACTGCGA	CTGCTTCCGCGCTCCTCAC

Supplemental Table 2: Primers for ChIP-PCR

Gene	Primer sequences (sense)	Primer sequences (antisense)
CDKN2A	ACAGGAGGGGAGCGGCCA	CTCTGACGCGACATCTGGACAC
BIRC3	GGGAAATATGGCAGTGCAATTAGACTCA	CTTCTACTATAGCTGCAGAAGTCCAGC
